# Fully-automatic synthesis of cine viability CMR images with minimal estimation error

**DOI:** 10.1186/1532-429X-17-S1-P106

**Published:** 2015-02-03

**Authors:** Azza S Hassanein, Ayman M Khalifa, El-Sayed Ibrahim

**Affiliations:** University of Michigan, Ann Arbor, MI USA; Helwan University, Cairo, Egypt

## Background

A typical CMR exam includes cine, tagging, and delayed-hyperenhancement (DHE) sequences to produce images for evaluating global heart function, myocardial contractility, and viability, respectively. Usually, DHE imaging is conducted at mid-diastole. Nevertheless, obtaining cine DHE images is appealing to obtain simultaneous information about tissue viability and wall motion abnormality. In this study, we compare the performance of four image analysis techniques for generating the cine DHE images based on estimating tissue motion, validate the results on numerical phantom, and implement on in-vivo images.

## Methods

A numerical phantom of a grid-tagged short-axis slice, that experiences cyclic deformation, was built to test the performance of four techniques for estimating the motion field used to generate the cine DHE images: harmonic phase (HARP)[1], and three optical-flow (OF) methods[2–4]: Lucas-Kanade optical-flow (LKOF), Horn-Shunck (HSOF), and band-pass (BPOF). The generated motion fields are compared to the ground-truth motion.

Cine, DHE, and tagged images were acquired from four patients with myocardial infarction (MI) on 3T scanner. The motion field was estimated from the tagged images and used to reconstruct cine DHE images starting from the acquired (known) DHE image. Wall thickening was measured from both cine and generated DHE images. MI was determined using full-width at half-maximum method. Circumferential strain was measured from the tagged images using Diagnosoft software.

## Results

**Figure 1:**
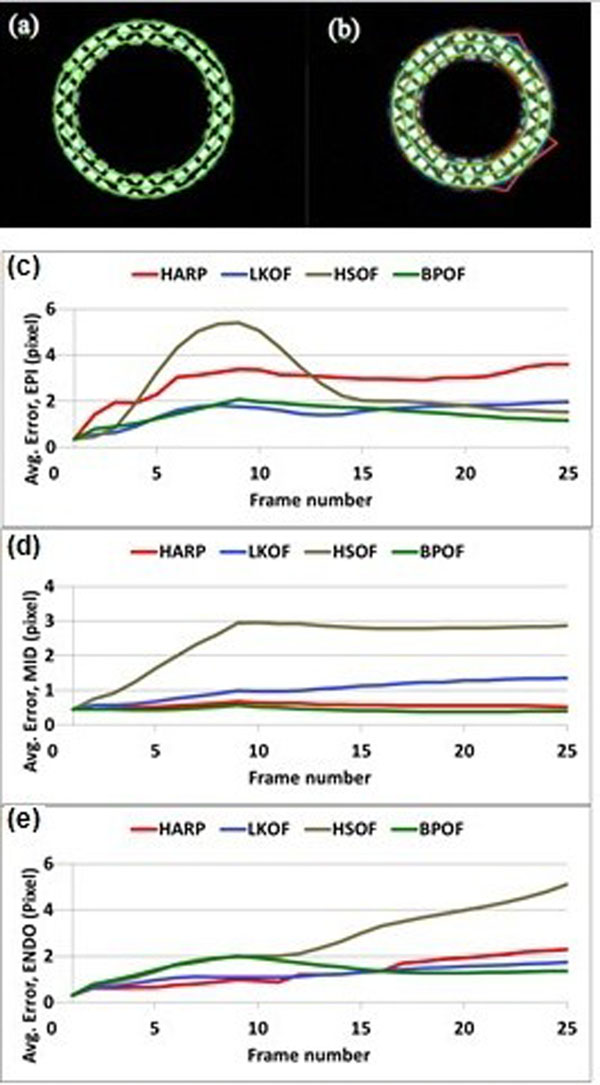
Phantom results. Grid-tagged images at (a) end-diastole and (b) end-systole, showing tracking results from BPOF (green) and HARP (red). Tracking error at (c) epicardium, (d) mid-myocardium, and (e) endocardium using HARP (red), LKOF (blue), HSOF (brown), and BPOF (green). BPOF shows lowest error amoung all methods, and outperforms HARP at the boundaries.

BPOF resulted in minimal tracking error compared to other techniques. The tracking accuracy of BPOF was close to HARP for intramyocardial points; however, it was significantly higher at the boundaries, where HARP failed to track the tag points. Fig.1(a,b) shows the tagging grid at end-diastole (initial frame) and tracking results at end-systole using HARP and BPOF. Fig.1(c-e) shows the average tracking error for points on the epicardium, endocardium, and mid-myocardium.

2 shows cine images, and corresponding synthesized DHE images (using BPOF), of the same slices at end-systole and end-diastole. The results showed strong correlation between wall thickening measured from both datasets (R>0.7). Further, myocardial strain showed strong inverse correlation (|R|>0.8) with MI transmurality measured from the synthesized DHE images.

**Figure 2:**
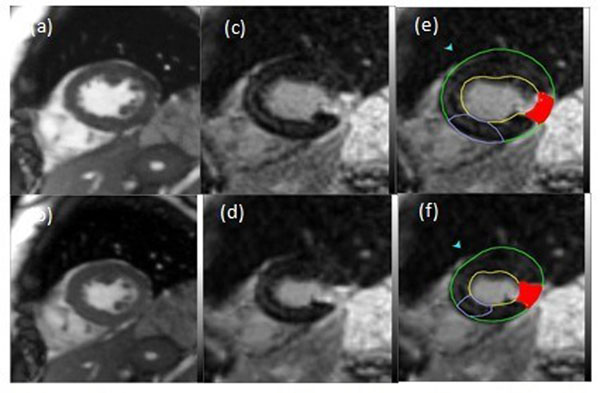
Cine images at (a) end-diastole and (b) end-systole with (c,d) corresponding synthetic DHE images. (e) and (f) show the DHe regions and wall thickness in corresponding synthetic DHE images.

## Conclusions

BPOF has minimal error in tracking taglines and measuring motion field, which can be used for generating cine DHE images. The generated cine DHE images may be important for comprehensive evaluation of the patient's condition, as they show simultaneous myocardial viability and wall motion abnormality on the same dataset without additional scan time or misregistration problems.

## Funding

N/A.
